# Proton Therapy for Head and Neck Cancer: A 12-Year, Single-Institution Experience

**DOI:** 10.14338/IJPT-20-00065.1

**Published:** 2021-06-25

**Authors:** G. Brandon Gunn, Adam S. Garden, Rong Ye, Noveen Ausat, Kristina R. Dahlstrom, William H. Morrison, C. David Fuller, Jack Phan, Jay P. Reddy, Shalin J. Shah, Lauren L. Mayo, Stephen G. Chun, Gregory M. Chronowski, Amy C. Moreno, Jeffery N. Myers, Ehab Y. Hanna, Bita Esmaeli, Maura L. Gillison, Renata Ferrarotto, Katherine A. Hutcheson, Mark S. Chambers, Lawrence E. Ginsberg, Adel K. El-Naggar, David I. Rosenthal, Xiaorong Ronald Zhu, Steven J. Frank

**Affiliations:** 1 Department of Radiation Oncology, University of Texas MD Anderson Cancer Center, Houston, TX, USA; 2 Department of Biostatistics, University of Texas MD Anderson Cancer Center, Houston, TX, USA; 3 Department of Head and Neck Surgery, University of Texas MD Anderson Cancer Center, Houston, TX, USA; 4 Ophthalmic Plastic Surgery, Department of Plastic Surgery, University of Texas MD Anderson Cancer Center, Houston, TX, USA; 5 Department of Medical Oncology, University of Texas MD Anderson Cancer Center, Houston, TX, USA; 6 Department of Neuroradiology, University of Texas MD Anderson Cancer Center, Houston, TX, USA; 7 Department of Pathology, University of Texas MD Anderson Cancer Center, Houston, TX, USA; 8 Department of Radiation Physics, University of Texas MD Anderson Cancer Center, Houston, TX, USA

**Keywords:** proton therapy, head and neck cancer, toxicity, survival

## Abstract

**Purpose:**

To characterize our experience and the disease control and toxicity of proton therapy (PT) for patients with head and neck cancer (HNC).

**Patients and Methods:**

Clinical outcomes for patients with HNC treated with PT at our institution were prospectively collected in 2 institutional review board–approved prospective studies. Descriptive statistics were used to summarize patient characteristics and outcomes. Overall survival, local-regional control, and disease-free survival were estimated by the Kaplan-Meier method. Treatment-related toxicities were recorded according to the Common Terminology Criteria for Adverse Events (version 4.03) scale.

**Results:**

The cohort consisted of 573 patients treated from February 2006 to June 2018. Median patient age was 61 years. Oropharynx (33.3%; n = 191), paranasal sinus (11%; n = 63), and periorbital tissues (11%; n = 62) were the most common primary sites. Patients with T3/T4 or recurrent disease comprised 46% (n = 262) of the cohort. The intent of PT was definitive in 53% (n = 303), postoperative in 37% (n = 211), and reirradiation in 10% (n = 59). Median dose was 66 Gy (radiobiological equivalent). Regarding systemic therapy, 43% had received concurrent (n = 244), 3% induction (n = 19), and 15% (n = 86) had both. At a median follow-up of 2.4 years, 88 patients (15%) had died and 127 (22%) developed disease recurrence. The overall survival, local-regional control, and disease-free survival at 2 and 5 years were, respectively, 87% and 75%, 87% and 78%, and 74% and 63%. Maximum toxicity (acute or late) was grade 3 in 293 patients (51%), grade 2 in 234 patients (41%), and grade 1 in 31 patients (5%). There were 381 acute grade 3 and 190 late grade 3 unique toxicities across 212 (37%) and 150 (26%) patients, respectively. There were 3 late-grade 4 events across 2 patients (0.3%), 2 (0.3%) acute-grade 5, and no (0%) late-grade 5 events.

**Conclusions:**

The overall results from this prospective study of our initial decade of experience with PT for HNC show favorable disease control and toxicity outcomes in a multidisease-site cohort and provide a reference benchmark for future comparison and study.

## Introduction

Advances in proton therapy (PT), principally planning and delivery of pencil-beam scanning intensity modulated PT (IMPT), have provided the ability to generate the obligate dose distributions to treat even the most geometrically complex tumors [1]. With these advances, and given the proximity of head and neck cancer (HNC) target volumes to numerous critical structures and avoidance organs, PT is increasingly selected because of the advantageous physical properties and dosimetry of protons compared with photons. The central goal of our PT program for HNC has been to improve patient outcomes through delivery of therapeutic doses to the target volumes and to minimize or even eliminate the unnecessary radiation dose to the surrounding nontarget healthy tissues and associated toxicities [2].

Comparative analyses have established that PT can achieve more optimal dose distributions compared with intensity-modulated radiation (x-ray) therapy (IMRT) for many HNC tumor sites, yet few prospective studies have reported clinical outcomes outside of individual studies reporting on subsites of specific interest [2]. Although we have previously reported our initial clinical experience and patient outcomes after PT for various HNC subsites [3–12] and a multi-institutional phase III randomized trial of IMPT versus IMRT in oropharyngeal carcinoma (ClinicalTrials.gov identifier: NCT01893307) is ongoing [13], comprehensive reporting of patient outcomes in large HNC cohorts are needed to inform the HNC and PT community.

To that end, the purpose of the present study was to (1) characterize our initial decade experience with PT for HNC, (2) report oncologic outcomes in a large prospective multi-subsite cohort, (3) report observed acute and late-treatment–related toxicities, and (4) provide benchmark data to generate testable hypotheses for future comparison and study.

## Patients and Methods

### Patients

Patients receiving PT for HNC cancer at our institution were eligible for participation in single-institution, institutional review board–approved registry studies where clinical outcomes were prospectively recorded. The first study encompassed all cancer types (ClinicalTrials.org identifier: NCT 00991094), whereas the subsequent study was HNC specific (ClinicalTrials.org identifier: NCT 01627093); both collected baseline and follow-up clinical data. Participants provided study-specific informed consent. No specified clinical criteria were used for patient selection for PT because all patients with HNC referred for radiation therapy at our institution were generally considered potential candidates for PT. The ultimate case-specific treatment decision for the use of PT was made according to physician clinical judgment, often supplemented by IMRT plan comparison, as part of a physician-patient shared decision-making process. The clinical selection of PT over IMRT plans was usually made according to improved or maintained coverage of target volumes, coupled with reduced doses to critical structures (eg, brain, brainstem, and optic structures) and/or other nontarget avoidance structures (eg, major or minor salivary glands, nontarget upper aerodigestive tract mucosa, and swallowing organs). Before therapy, patients underwent staging imaging, pathologic confirmation of malignancy, and multidisciplinary evaluation, and all cases were presented at our HNC multidisciplinary tumor board for personalized consensus-treatment recommendations.

For this analysis, we considered the initial cohort of consecutive study participants with HNC (including cutaneous cancers involving the head and neck) treated at our center who received either three-dimensional passive scatter PT (PSPT) and/or IMPT. Those treated on a phase III oropharynx randomized trial (ClinicalTrials.gov identifier: NCT01893307) were excluded from this analysis. Patient follow-up, outcomes, and toxicity assessments were performed as described previously [3]. Briefly, at each in-person visit, treatment-related toxicity endpoints were assessed by the treating radiation oncologist according to the Common Terminology Criteria for Adverse Events (version 4.03) scale. Acute toxicities were defined as those observed between the start of PT and within 90 days of completion, whereas late toxicities were defined as those observed > 90 days after completion.

### Treatment

Our general treatment philosophies for HNC, including integration of systemic therapy, radiation therapy dose and fractionation, and target volume delineation [14], and the technical specifications of our PT system [15] have been described previously. Regarding treatment simulation for PT, patients underwent noncontrast computed tomography (CT) simulation in the supine position and were immobilized with a customized posterior head, neck, and shoulder mold or cradle, a full-length thermoplastic mask, and a bite block with or without a customized oral stent. In more recent years, emphasis had been placed on ensuring the mold and mask supported a more reproducible and neutral neck position (rather than overly extended) with reinforcement over the apex of the shoulders to improve daily setup reproducibility. Before treatment planning, each patient underwent a physical examination, including fiberoptic endoscopic examination, as appropriate. The proposed target volumes were prospectively peer reviewed before treatment planning at our Head and Neck Radiation Oncology Planning and Development Clinic for quality assurance (QA) purposes [16].

The PT doses were prescribed using a relative biological effectiveness (RBE) value of 1.1. In the definitive setting, 66 Gy(RBE) in 30 to 33 daily fractions or 70 Gy(RBE) in 33 to 35 fractions was generally prescribed for small-volume disease and more advanced disease, respectively. In the postoperative setting, 60 Gy(RBE) in 30 fractions or 63 to 66 Gy(RBE) in 30 to 33 fractions was prescribed. For IMPT plans, a simultaneous integrated boost technique was used in which lower daily doses were delivered to elective regions at risk of harboring microscopic disease and were typically prescribed 54 to 63 Gy(RBE), depending on the estimated risk and prescribed number of fractions. The PSPT plans were generally treated with a sequential, shrinking-field technique, with a daily dose of 2 Gy(RBE), ensuring a field size > 2 × 2 cm. In the reirradiation setting, 66 Gy(RBE) in 33 fractions was generally prescribed in the definitive setting, 60 Gy(RBE) in 30 fractions postoperatively, and target volumes were generally restricted to gross disease or the tumor bed with a margin without reirradiation of elective regions [9].

The PT plans were generated in the Eclipse treatment planning system (Varian Medical Systems, Palo Alto, California) and typically consisted of 2 to 6 beams, depending on the location and the extent of target volumes. Generally, 3 beams were used for bilateral neck IMPT plans, with a left and right anterior-inferior oblique and a posterior beam [1]. The IMPT treatment plans were mainly optimized with multifield optimization (since 2010), with the objective of covering ≥ 95% of the target volumes with the prescribed doses and minimizing and balancing the dose among nontarget structures. The dose constraints considered for avoidance organs were generally photon derived, but PT plans were optimized well below traditional constraints, with the goal of realizing the maximal dosimetric and clinical benefit of PT. The proton spot size at the isocenter ranged from 5 mm to 14 mm in the air. For patients with unilateral neck treatment, PSPT or IMPT with single-field optimization was also considered. The robustness of each beam and treatment plan was considered, and all IMPT plans were evaluated by robust evaluation since 2014, with implementation of robust optimization in 2017, with both approaches accounting for variations in patient setup (3 mm in every direction) and proton range (± 3.5%) [17]. Reduced distal margins were considered to ensure protection of critical structures, as necessary.

Before PT delivery, plan-specific QA was performed, which included independent dose calculation, field measurements, and analysis of delivery log files for IMPT plans and compensator and aperture QA, and field measurements for PSPT plans [18]. Orthogonal 2-dimensional kilovolt x-ray images were used for daily image guidance. Patients underwent verification CT simulation during weeks 1 and 4 of PT (or more frequently as needed) to determine the effect of anatomic changes (eg, weight loss or tumor regression) on PT dose distributions. More recently, in addition to CT simulation, initial and verification magnetic resonance simulations, with or without contrast, were commonly performed to assist in target volume and organ-at-risk delineation. Likewise, weekly verification CT simulations have recently been performed for cases with paranasal sinus or skull base targets to detect random changes in aeration or opacification of sinuses that could affect target coverage and/or dose to critical structures. In general, adaptive replanning was considered on a case-by-case basis and performed at the judgment of the treating physician after review of the verification PT plan dose distributions and in consultation with our clinical PT physicists [19].

### Statistical Analysis

Descriptive statistics were used to summarize patient demographic, tumor and treatment characteristics, and toxicities. Primary tumor sites with < 15 patients were grouped as *other*. Periorbital tumors were primaries of the lacrimal gland, the nasolacrimal apparatus, and the eyelids. Follow-up time was calculated by the reverse Kaplan-Meier method. The distribution of time-to-event endpoints from the end of PT for overall survival (OS), local-regional control (LRC), and disease-free survival (DFS) were estimated with the Kaplan-Meier method, and group-survival differences were determined with the log-rank test. All tests were 2 sided, and *P* < .05 was considered statistically significant. Statistical analyses were performed with Stata 16.0 (StataCorp, College Station, Texas).

## Results

### Patient, Tumor, and Treatment Characteristics

Five hundred and seventy-three patients treated from February 2006 to June 2018 formed the cohort. The patient and tumor characteristics for the overall study cohort are shown in Table 1. Median patient age was 61 years (interquartile range, 49–68). The most common histologies were squamous carcinoma in 340 patients (59.3%) and adenoid cystic carcinoma in 74 patients (12.9%). One hundred and sixty-one of the 174 oropharyngeal tumors (92.5%) tested were positive for *p16*. Overall, 45.7% patients (n = 262) were treated for T3/T4 or recurrent disease, and 60.5% patients (n = 347) had stage IV disease (AJCC, 7th edition [20]). Two hundred and eighty-six patients (49.9%) were never smokers, 34 (5.9%) were current smokers, and 233 (40.7%) were former smokers, whereas 20 patients (3.5%) had an unknown smoking status.

**Table.** Patient and tumor characteristics of the overall study cohort.

**Parameter**	**No. (%), N = 573**
Sex	
Men	398 (69.5)
Women	175 (30.5)
Race	
White	466 (81.3)
Other/unknown	46 (8.0)
Black/African American	30 (5.2)
Asian	28 (4.9)
Native American	3 (0.5)
Primary site	
Oropharynx	191 (33.3)
Paranasal sinus	63 (11.0)
Periorbital	62 (10.8)
Parotid	50 (8.7)
Nasopharynx	49 (8.6)
Other	45 (7.9)
Oral cavity	38 (6.6)
Cutaneous	36 (6.3)
Larynx	21 (3.7)
Base of skull	18 (3.1)
T-category^a^	
T0	13 (2.3)
Tx	68 (11.9)
T1	107 (18.7)
T2	123 (21.5)
T3	42 (7.3)
T4	106 (18.5)
Recurrent	114 (19.9)
N category^a^	
N0	154 (26.9)
Nx	66 (11.5)
N1	66 (11.5)
N2	165 (28.8)
N3	9 (1.6)
Recurrent	113 (19.7)
Proton therapy treatment, y	
2006-09	4 (0.7)
2010	6 (1.0)
2011	33 (5.8)
2012	71 (12.4)
2013	58 (10.1)
2014	66 (11.5)
2015	94 (16.4)
2016	87 (15.2)
2017	98 (17.1)
2018 (through June)	56 (9.8)

a AJCC 7th Edition [20].

Three hundred and three patients (52.9%) received definitive PT, 211 (36.8%) received postoperative PT, and 59 (10.3%) received PT for reirradiation (either definitive or postoperative). The most common disease sites for the definitive, postoperative, and reirradiation treatment groups were oropharynx (51.2%; 155 of 303), periorbital or paranasal sinus (40.3%; 85 of 211), and oropharynx (27.1%; 16 of 59), respectively.

Regarding PT modality, 67.5% (n = 387) of patients received IMPT exclusively, 15.5% (n = 89) received 3-dimensional PSPT exclusively, 14.8% (n = 85) received a combination of IMPT and PSPT, and 2.1% (n = 12) received PT in combination with IMRT. The initial patients in this cohort were treated with PSPT, and the first patient with IMPT was treated in 2008. Median PT dose was 66 Gy(RBE), and median dose per fraction was 2 Gy(RBE). Regarding systemic therapy, 244 patients (42.6%) received concurrent therapy, 19 (3.3%) received induction therapy, and 86 (15.0%) received both induction and concurrent therapy. Induction regimens were generally platinum and taxane based. Concurrent regimens were typically a single agent, and those patients who received concurrent therapy (n = 330) were treated with cisplatin (195 of 330; 59.1%), carboplatin (76 of 330; 23.0%), or cetuximab (43 of 330; 13.0%).

### Survival and Disease Control

**Figure 1.**
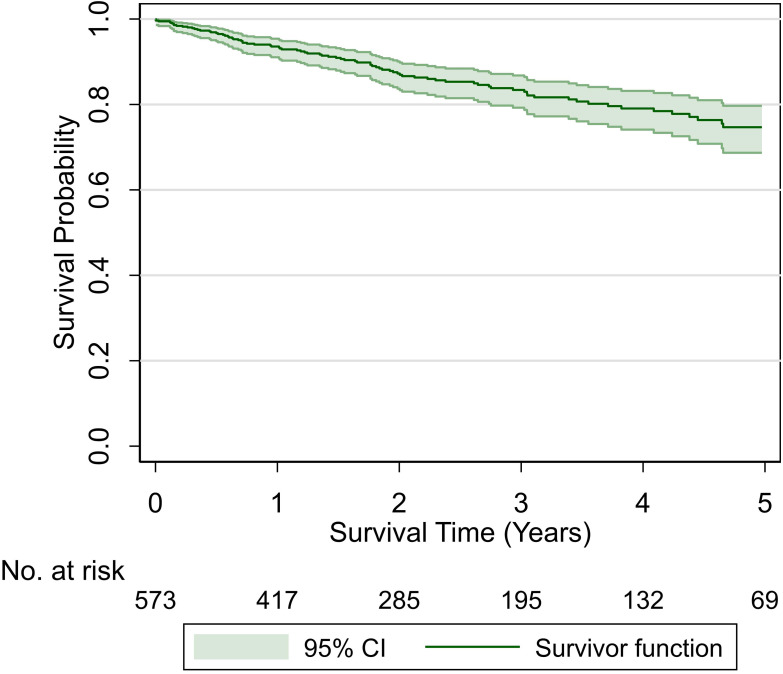
Overall survival and 95% CI after proton therapy for head and neck cancer for the entire study cohort.

At a median follow-up of 2.4 years (interquartile range, 1.1–4.4), 88 patients (15.3%) had died, including 50 of 88 (56.8%) who had active HNC at death. One hundred and twenty-seven patients (22%) had disease recurrence, which was local-regional in 82 (64.6%) and distant in 45 (35.4%) of the 127 patients. The estimated OS (with 95% CI) for the overall study cohort is shown in Figure 1. The estimated OS, LRC, and DFS at 2 and 5 years, respectively, for the overall study cohort were 87% and 75%, 87% and 78%, and 74% and 63%.

**Figure 2.**
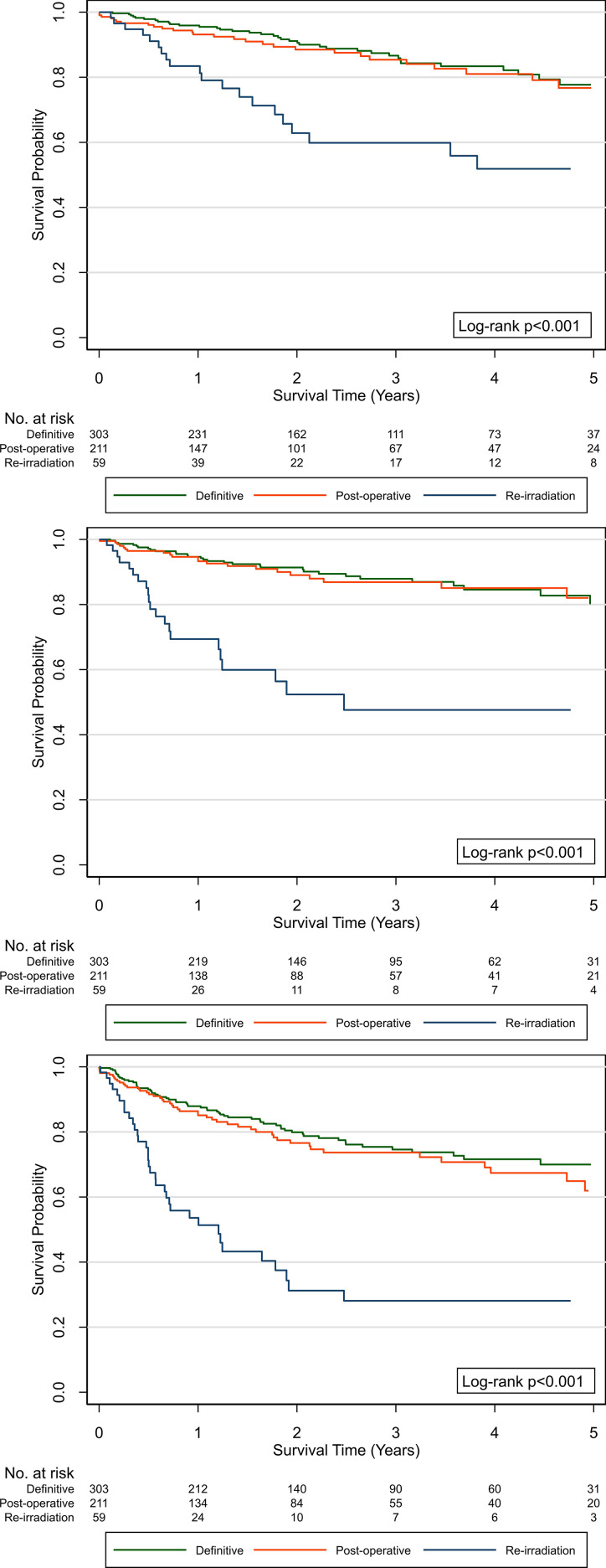
Overall survival (top), local-regional control (middle), and disease-free survival (bottom panel) after proton therapy for head and neck cancer, by treatment group.

**Figure 3.**
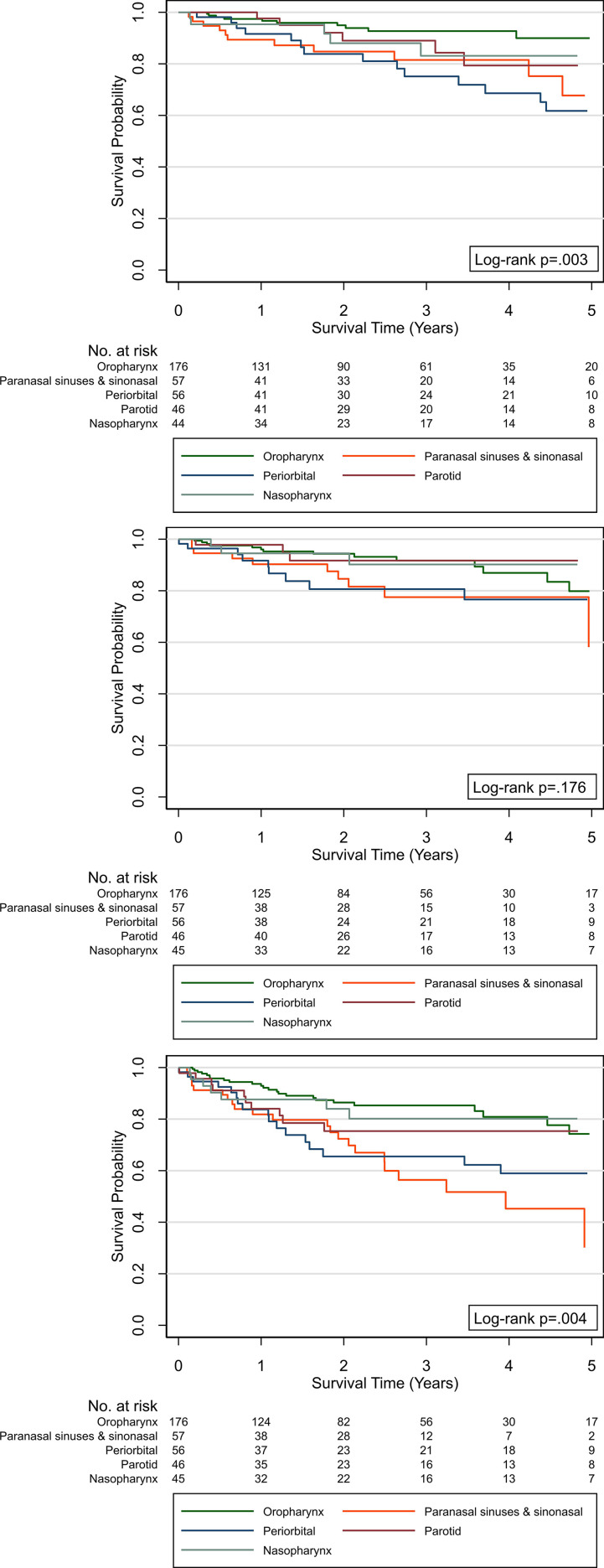
Overall survival (top), local-regional control (middle), and disease-free survival (bottom) after definitive or postoperative proton therapy for the top five most commonly treated sites, by primary site.

The estimated OS, LRC, and DFS by treatment group are shown in Figure 2. The OS at 2 and 5 years by treatment group was, respectively, 91% and 78% (definitive), 89% and 77% (postoperative), and 63% and 52% (reirradiation). The LRC at 2 and 5 years by treatment group was 91% and 80% (definitive), 89% and 82% (postoperative), and 52% and 48% (reirradiation). The DFS at 2 and 5 years by treatment group was 80% and 70% (definitive), 77% and 62% (postoperative), and 31% and 28% (reirradiation). The estimated OS, LRC, and DFS for the top 5 most-common primary sites treated with definitive or postoperative PT (reirradiation treatment group excluded) are shown in Figure 3.

### Toxicity

Maximum treatment-related toxicity (acute or late) was grade 3 in 293 patients (51.1%), grade 2 in 234 patients (40.8%), and grade 1 in 31 patients (5.4%). There were 381 acute grade 3 unique toxicities across 212 patients (37%). Eighty-eight patients (15.4%) had a feeding tube placed or present during PT; of which, 46 (52.3%) had the tube removed at last follow-up, with a median tube duration of 83 days. The most-frequent grade 2^+^ acute toxicities observed were radiation dermatitis in 482 patients (84.1%), mucositis in 314 patients (54.8%), and dysphagia in 137 (23.9%). There were no (0%) acute grade 4 toxicities. There were no (0%) acute grade 5 events during PT. There were 2 grade 5 events (0.3%), which occurred within 90 days from the end of PT: 1 patient who underwent reirradiation with concurrent chemotherapy for oropharyngeal cancer died from septic shock and multiorgan treatment failure, likely secondary to pneumonia; and the other patient underwent postoperative PT for oral cavity cancer and died from a traumatic cause.

There were a total of 190 late-grade 3 unique toxicities across 150 patients (26.2%). The most frequent grade 2^+^ late toxicities observed were xerostomia in 169 patients (29.5%), radiation dermatitis in 162 patients (28.3%), and dysgeusia in 100 patients (17.5%). There were 3 late grade 4 unique toxicities across 2 patients (0.3%). All 3 of these events were ocular and included corneal abrasion, retinopathy, and optic neuropathy. The former 2 were managed and improved with medical and surgical therapy, whereas the latter resulted in mono-ocular loss of vision. The latter was anticipated because the tumor encased the optic nerve. There were no late grade 5 events.

## Discussion

The overall disease control and acute and late toxicities after PT in this mixed cohort of nearly 600 HNC patients were largely favorable, which further supports PT as a standard and effective treatment option for HNC and our clinical philosophies in terms of patient selection and PT planning and delivery. Notably, these outcomes were achieved despite nearly one-half of patients having been treated for locally advanced (T3/T4; 262 of 573; 46%) or recurrent primary tumors and 60% (349 of 573; 61%) having also received systemic therapy. This suggests that these outcomes were not likely achieved through selection of patients with anticipated favorable disease control or toxicity outcomes per se but, more likely, were encouraging outcomes, despite the typical HNC patient and tumor complexity managed at tertiary centers that may be preferentially referred for PT, such as patients with more locally advanced disease and/or uncommon or particularly challenging tumor locations (eg, those in immediate proximity to dose-limiting critical structures).

Although the oropharynx was the most represented disease site, consistent with the overall recent epidemiologic trends in HNC, we elected to include all disease sites in this analysis, including mucosal and nonmucosal primary sites and patients receiving reirradiation to demonstrate our practice patterns and application of PT technology. We also analyzed disease control in definitive and postoperative PT groups separately because patients treated in the postoperative setting can have distinct anatomic changes and tissue or reconstruction hardware heterogeneity, which can add to the complexity of initial PT planning. Likewise, for patients treated definitely, interfractional anatomic changes across the course of treatment, such as tumor regression or progression and weight loss, can negatively affect PT dose distributions, which must be monitored with PT plan verification and addressed with adaptive planning as needed. Despite those complexities, the LRC rates in the postoperative group and definitive group were largely favorable and generally comparable.

We have previously detailed patient and treatment characteristics, disease control, and toxicity outcomes for patients treated at our center with PT for oropharyngeal cancer [3, 10, 12, 21–23], skull base tumors [24], periorbital tumors [5], nasopharyngeal cancer [4], adenoid cystic carcinomas [6, 7], and paranasal sinus tumors [11] and for patients treated with PT for reirradiation [8, 9, 25]. Evidence from initial comparative reports in oropharyngeal cancer from our center and others, for example, have demonstrated reduced acute toxicities [26], reduced weight loss, reduced feeding tube placement [22], reduced symptom severity during the subacute recovery period [21], reduced incidence of osteonecrosis [23], and no difference in overall survival with IMPT compared with IMRT [22]. We have also recently demonstrated lower than anticipated total medical cost for patients with cancer treated with PT, which compared favorably to those treated with IMRT in a prospective, case-matched insurance-coverage pilot study [27]. Based on these encouraging disease control rates, toxicity profiles, and the potential value proposition of reduced toxicities with PT, a prospective, multi-institutional randomized phase III trial comparing IMPT and IMRT for patients with locally advanced oropharyngeal carcinoma is being conducted (ClinicalTrials.gov identifier: NCT01893307), anticipated to complete accrual in 2021 [13], and we continue to prioritize eligible patients for enrollment on this trial at our center.

Presently, approximately 30% of patients with HNC treated with radiation therapy at our institution receive PT, and patients with HNC have made up approximately 30% to 40% of all patients treated at our PT center in recent years. Moreover, PT therapy remains a preferred approach for most patients at our center with skull base (including nasopharynx), paranasal sinus, and periorbital tumors and for patients referred for unilateral neck radiation therapy. Given the obligation to minimize the overlap of current and previous doses to minimize the risk of high-grade toxicity, PT remains an important treatment consideration for reirradiation at our center. A randomized phase II reirradiation study is being conducted at our center comparing the toxicity of stereotactic ablative radiotherapy (x-ray) versus fractionated IMRT or IMPT for patients with unresectable HNC (ClinicalTrials.gov identifier: NCT03164460).

Although this is, to our knowledge, the largest study to report outcomes of patients with HNC after PT, the inherent limitations of a single-institution series do apply. It is not possible to fully characterize, in this report, the iterative technical improvements and gain in expertise that occurred across the learning curve during our initial 12 years of experience. Although we did not focus on site-specific outcomes in this report, as with our previous site-specific studies, future studies should incorporate baseline and longitudinal patient-reported outcomes, correlative dosimetric factors (including exploratory correlation with linear energy transfer and variable RBE modeling), development of PT-specific dose constraints and clinical decision tools, such as healthy tissue complication probability modeling, and serial objective measures of toxicity and function (eg, weight loss, swallowing function, and feeding tube placement) most relevant for each specific site. Most patients in this cohort were treated in the more-recent years, and patients may opt for long-term follow-up closer to home, rather than at a distant tertiary center, which affected the median follow-up time in our study cohort. Longer follow-up is needed (ideally, > 5 years) to substantiate these disease control and survival rates and for late toxicities, in particular.

In conclusion, the overall results from this prospective study of our initial decade of experience with PT for HNC show favorable disease control and toxicity outcomes in a multi-disease site cohort in the definitive, postoperative, and re-irradiation setting and provide a reference PT benchmark for additional comparison and future study.
